# Effects of Chitin and Sepia Ink Hybrid Hemostatic Sponge on the Blood Parameters of Mice

**DOI:** 10.3390/md12042269

**Published:** 2014-04-10

**Authors:** Wei Zhang, Yu-Lin Sun, Dao-Hai Chen

**Affiliations:** 1 Zhanjiang Normal University, Round Beibu Gulf Institute for the Protection and Utilization of Marine Animals in Medicine, Zhanjiang 524048, China; E-Mails: khzhangwei110@sina.com (W.Z.); sunyulin07002@126.com (Y.-L.S.); 2 Life Science and Technology School, Zhanjiang Normal University, Zhanjiang 524048, China

**Keywords:** chitin, sepia ink, coagulation, anticoagulation, fibrinolytic, hemorheology

## Abstract

Chitin and sepia ink hybrid hemostatic sponge (CTSH sponge), a new biomedical material, was extensively studied for its beneﬁcial biological properties of hemostasis and stimulation of healing. However, studies examining the safety of CTSH sponge in the blood system are lacking. This experiment aimed to examine whether CTSH sponge has negative effect on blood systems of mice, which were treated with a dosage of CTSH sponge (135 mg/kg) through a laparotomy. CTSH sponge was implanted into the abdominal subcutaneous and a laparotomy was used for blood sampling from abdominal aortic. Several kinds of blood parameters were detected at different time points, which were reflected by coagulation parameters including thrombin time (TT), prothrombin time (PT), activated partial thromboplatin time (APTT), fibrinogen (FIB) and platelet factor 4 (PF4); anticoagulation parameter including antithrombin III (AT-III); fibrinolytic parameters including plasminogen (PLG), fibrin degradation product (FDP) and D-dimer; hemorheology parameters including blood viscosity (BV) and plasma viscosity (PV). Results showed that CTSH sponge has no significant effect on the blood parameters of mice. The data suggested that CTSH sponge can be applied in the ﬁeld of biomedical materials and has potential possibility to be developed into clinical drugs of hemostatic agents.

## 1. Introduction

Chitin is a homopolymer of 1–4 linked 2-acetamido-2-deoxy-β-D-glucopyranose, although some of the glucopyranose residues are deacetylated and occur as 2-amino-2-deoxy-β-D-glucopyranose [1], in addition, chitin is the only alkaline polysaccharide in nature [2]. Chitin and its derivatives are biodegradable and biocompatible biomedical materials to humans and most animals [3]. Previous reports have indicated that there are numerous studies and applications about chitin in hemostatic materials, drug delivery and tissue repairing materials [4,5,6,7,8]. The hemostatic mechanism of chitin includes two methods. One is that chitin and platelet are adhered to each other through protein-mediation, then the composite of chitin/platelet is formed, which can accelerate the polymerization of fibrin monomers and form clots together. In the other, chitin induces erythrocyte aggregation and stimulates vasoconstriction. Ultimately, thrombosis is formed and the wound is sealed [9].

Sepia ink is mainly composed of melanin [10], and some researchers have founded that sepia ink has anti-radiation activity, antitumor activity, immunomodulatory activity, procoagulant function, *etc*. [11]. Degradation studies on sepia melanin show that it is a copolymer of 5,6-dihydroxyindole (DHI) and 5,6-dihydroxyindole-2-carboxylic acid (DHICA), which are postulated to be the main monomeric building blocks of eumelanin [12]. Sepia ink is insoluble in water and organic solvents by either chemical method or enzymatic hydrolysis, because of its insolubility over a broad range of pH, but alkaline solution. It maintains a stable nature for many physical and chemical treatment methods [13].

Sepia ink had been used in the treatment of hemostasis for centuries in Chinese traditional medicine. As a kind of hemostatic drug, it was first used to treat heart pain listed in the Compendium of Materia (Medica compiled by Shizhen Li, a famous doctor at the time of the Ming Dynasty). Modern clinical medicine has proven that it is a good hemostatic medicine that also provides significant curative effects in gynecology, surgery, *etc*., [14]. The Institute of Oceanology, Chinese Academy of Sciences (Qingdao, China) had proved that there was obvious recent hemostatic efficacy for 400 cases of women, dysfunctional uterine bleeding patients in clinical trials who took the capsules of sepia ink, with an effective rate from 82.6% to 89.6%. For the gastrointestinal bleeding and long-term spotting tuberculosis bleeding, there are also good effects, and improvements in the functional status of the whole body [15].

Considering the good hemostatic effect and biocompatibility of chitin and sepia ink, the chitin and sepia ink hybrid sponge (CTSH sponge) was manufactured to combine their advantages. Various types of hemostatic agents or sponges have been explored, such as light-cured gelatin hydrogel glues, cyanoacrylate adhesives, microcrystalline collagen and collagen sponges, gelatin sponge, fibrin glue [16,17,18]. Unfortunately, each of them has its own shortcomings. For example, the collagen, fibrinogen or thrombin are mainly obtained from animal or human blood, which are expensive and include the potential risk of viral infection. The adhesive properties of collagen and gelatin sponges to tissues are poor. Water-soluble collagen is a toxicoid, the use of this material in the human body may cause inflammatory responses [19]. Therefore, it is meaningful to develop safe and new biological hemostatic agents for clinical use with better hemostatic properties, adhesion and reduced tissue response. CTSH sponge, a novel hemostatic materials, can be completely degraded in the abdominal cavity of mice about 160 days later observed in the process of extracting the blood from abdominal aortic of mice. The dosage is 135 mg/kg which was calculated from the mount of human about 1500 mg/70 kg [20]. Biological safety is important for the long term development of absorbable biomedical materials. It is necessary to detect whether CTSH sponge has signiﬁcant effects on the coagulation and ﬁbrinolytic functions after absorption and degradation in the blood system. The results will help to determine whether this biomedical material can be used safely as a potential therapeutic drug for humans.

In this study, CTSH sponge was prepared for its good biocompatibility and hemostasis. Compared with most other hemostatic sponges, CTSH sponge is a pure biological product, which only contains chitin and sepia ink; any other chemical agents including crosslinkers do not exist. The raw materials of CTSH sponge are cheap and easy to get. CTSH sponge has good mechanical properties and can effectively absorb excess exudates slowly, so it could keep the wound surface relatively dry and provide a moist microenvironment for wound healing. The wound bed would be covered completely, so the wound area would be well prevented the friction from outside, like clothing. Preliminary observation found that there was inflammatory response during the degradation time and no cytotoxicity. On the other hand, CTSH sponge has a sticky surface after absorbed body fluid, together with the extremely soft property, so it would adhere to the wound surface tightly, reducing the amount of bleeding and cutting down the bleeding time. In the degradation process of CTSH sponge, there was nearly no debris observation; deep tissues such as bone, tendon and nerve are sufficiently protected [21]. The basic properties of CTSH sponge were determined in this experiment, such as color, ash content, water absorption and deacetylation degree. The main purpose of this study was to elucidate coagulation, anticoagulant, fibrinolytic and hemorheology of mice with CTSH sponge over time and to detect whether these parameters were affected. This is the first time the effects of CTSH sponge have been reported in the biomedical materials ﬁeld.

## 2. Results and Discussion

At the end of their observation period, there was no obvious pathological change in any of the experimental animals at necropsy. None of the experimental animals died during the observation period and no abnormal behavioral changes were observed comparing to the control group.

### 2.1. Properties of Chitin and Sepia Ink Sponge

The basic properties of CTSH sponge used in this experiment are showed in Table 1. CTSH sponge appears gray, and its water absorption is up to 25 times within 3 s. According to the Chinese Pharmacopoeia of 2005 edition, the ash content was detected as 1.224%, which complies with the safety regulations of biomedical materials. Then, the deacetylation degree obtained from acid-base titration is 30.700%.

**Table 1:** Properties of chitin and sepia ink hybrid hemostatic (CTSH) sponge.

Color	Water absorption (/time)	Ash content (%)	Deacetylation degree (%)
gray	25	1.224	30.700

As a biodegradable hemostatic sponge, CTSH sponge can be prepared for different sizes and shapes according to actual needs. The CTSH sponge is more suitable for the relatively regular wound bed, both *in vivo* and *in vitro*, because the exudates, including blood, also ooze slowly.

### 2.2. Effects of CTSH Sponge on the Coagulation Parameters

**Figure 1:**
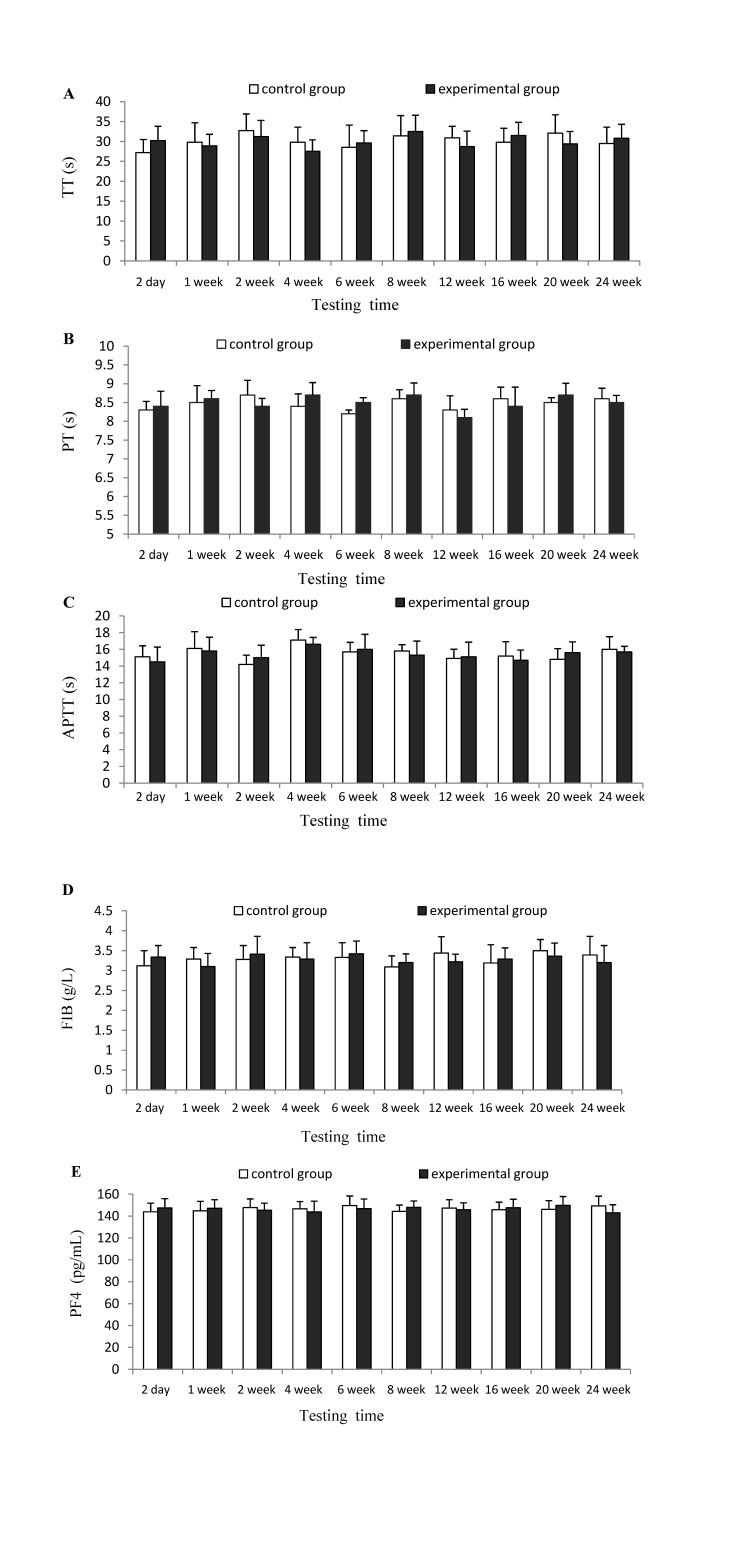
Effects of CTSH sponge on the coagulation parameters including (**A**) thrombin time (TT), (**B**) prothrombin time (PT), (**C)** activated partial thromboplatin time (APTT), (**D**) fibrinogen (FIB) and (**E**) Platelet factor 4 (PF4), *p* > 0.05 at each testing time.

As shown in Figure 1, results found that there was no significant (*p* > 0.05) difference between experimental group and control group at each testing time for TT, PT, APTT, FIB and PF4 indexes, which indicates that CTSH sponge had no significant effects on the coagulation functions of mice.

### 2.3. Effects of CTSH Sponge on the Anticoagulation Parameters

**Figure 2:**
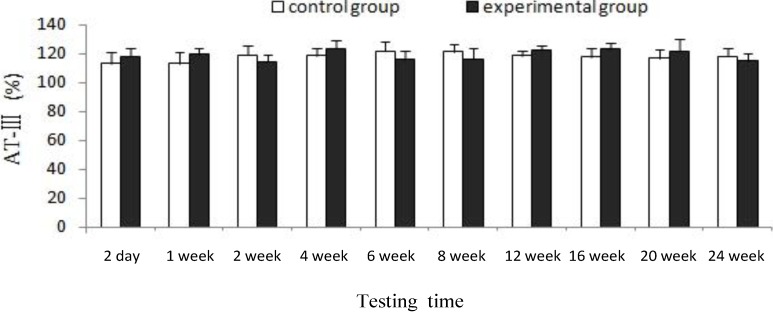
Effects of CTSH sponge on the coagulation parameter of AT-Ⅲ, *p* > 0.05 at each testing time.

Figure 2 illustrated that both experimental and control group showed much the same coagulation indicator, there was no significant difference (*p* > 0.05) for AT-III between experimental group and control group at each testing time, which indicates that CTSH sponge had no significant effects on the anticoagulation functions of rats after being absorbed in the abdominal cavity and degraded in the blood.

### 2.4. Effects of CTSH Sponge on the Fibrinolytic Parameters

**Figure 3:**
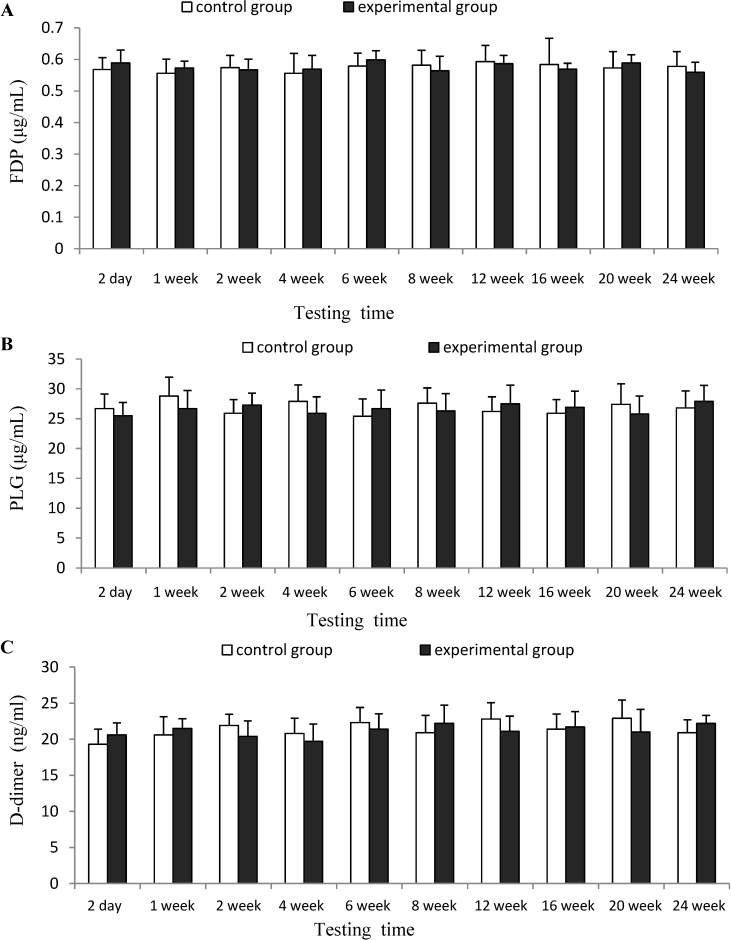
Effects of CTSH sponge on the fibrinolytic parameters including (**A**) (FDP), (**B**) (PLG) and (**C**) (D-dimer), *p* > 0.05 at each testing time.

The results of Figure 3 suggested that there was no significant difference (*p* > 0.05) for the indexes of FDP and FLG between experimental group and control group at each testing time. Therefore, CTSH sponge was considered to have no significant effects on the fibrinolytic function of mice after being absorbed in the blood.

### 2.5. Effects of CTSH Sponge on the Hemorheology Parameters

**Figure 4:**
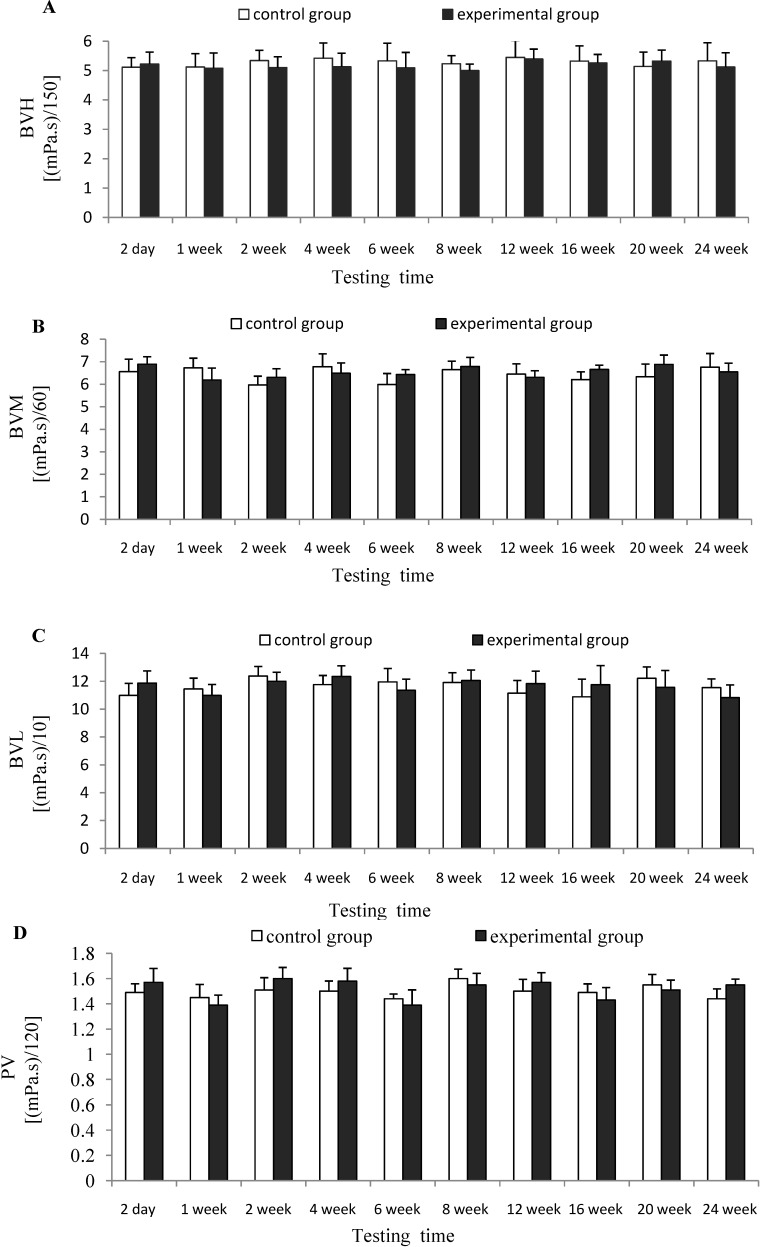
Effects of CTSH sponge on the hemorheology parameters including (**A**) (BVH), (**B**) (BVM), (**C**) (BVL) and (**D**) (PV), *p* > 0.05 at each testing time.

Figure 4 depicted that there was no significant difference (*p* > 0.05) for the indexes of BVL, BVM, BVH and PV between experimental group and control group at each testing time, which indicates that CTSH sponge had no significant effects on the hemorheology of rats after being absorbed *in vivo*.

Previous studies indicated that chitin sponge had achieved good hemostatic effects. In addition to hemostasis, the advantages of chitin sponge included pain relief, adherence and drying [21]. In comparison with the regular type of chitin, the sponge showed no autolysis or breaking and have possibilities for long term use in wound with extensive exudate. No negative effects were observed [21]. In this experiment, the safety of CTSH sponge, as a new kind of hemostatic material, was very necessary to determine. It is related to the possibility of the clinical application of this material. Therefore, this study has clarified whether and how long this material would affect the blood system in the process of degradation and after it completely was degraded *in vivo*.

To achieve hemostasis, the process of coagulation and fibrinolysis is exquisitely regulated by a variety of mechanisms, including initiators, cofactors, feedback reactions, and inhibitors. PT is a sensitive and commonly used screening test for the extrinsic coagulation system, the content of which reflects the overall activity of coagulation factor II, VII, IX, and X in plasma [22]; APTT is a sensitive and commonly used screening test for endogenous coagulation system, the content of which reflects the level of coagulation factor V, VIII, IX, XI, and XII in plasma [23]; TT primarily reflects whether there is an abnormal level of fibrinogen, anticoagulant and fibrinolytic substance in the common pathway of coagulation process that fibrinogen converted to fibrin [24]. FIB (also named clotting factor I) is an acute phase protein during the process of coagulation [25]. PF4, which is also known as CXCL4, is released from alpha-granules of activated platelets and binds with high affinity to heparin-like molecules promoting coagulation [26]. PLG is mainly produced by liver and endothelial cells, the content of it can reflect the fibrinolytic activity [27]. FDP is produced through the degradation of fibrin and fibrinogen, and the amount of which could be enhanced by primary and secondary hyperfibrinolysis [28]. D-dimer is a peptide fragment degraded by crosslinked fibrin which reflects the fibrinolytic activity more specifically than FDP. Studies have regarded the higher plasma D-dimer concentration as a sign of a hypercoagulable *in vivo* [29]. In this study, we found that CTSH sponge had no significant effects on the coagulation and fibrinolysis parameters of mice after being absorbed and degraded gradually *in vivo* during the testing time.

The determination of anticoagulant function mainly refers to the AT-III which is a serine protease inhibitor synthesized by the liver [30]. AT-III is the most important physiological anticoagulant; it can maintain the balance of coagulation and anticoagulation *in vivo* by inhibiting thrombin and other clotting factor activity [31]. Coagulation is also controlled by an anticoagulant pathway composed of natural antithrombotic factors that serve to put brakes on specific points in the coagulation cascade, which when left unchecked could lead to massive thrombosis [23]. In this study, the content of AT-III was detected to evaluate the effects of CTSH sponge on the anticoagulation performance of mice. Results showed that there was no significant difference between experimental and control groups at each testing time point, which suggested that CTSH sponge had no significant effects on the anticoagulation performance of mice after being absorbed *in vivo*.

The whole blood and plasma viscosity are important determinants of hemorheology in clinical examination [32]. BVH mainly generated from the red blood cell deformability. Higher BVH can cause the red blood cell deformability or poor flexibility and more fibrinogen is transformed into fibrin [33]. BVM is a transition point that BVL changes to BVH. BVL is mainly determined by the aggregation of red blood cells. Higher BVL can increase the aggregation of red blood cells. PV mainly depends on the level of plasma proteins, especially the concentration of fibrin [32]. The aim of this experiment was to evaluate whether CTSH sponge would affect the contents of blood and plasma viscosity. Our findings showed that CTSH sponge had no significant effects on hemorheology of mice after being absorbed *in vivo*.

## 3. Experimental Section

### 3.1. Materials and Regents

Chitin (β-chitin) acetylated 95% purchased from Qingdao Biotemed Biomaterimal Co., Ltd. (Qingdao, China). Fresh cuttlefish (*Sepia pharaonis* Ehrenberg) were purchased directly from Nanhua fish market of Zhanjiang city and rapidly transferred to the laboratory, then ink sacs were picked and stored at 4 °C before use. 120 adult Wistar mice (200 ± 20 g) were purchased from Jun Ke Biological Engineering Co., Ltd. (Qingdao, China). Platelet factor 4 (PF4) and fibrin degradation product (FDP) and plasminogen (PLG) ELISA kits were purchased from Adlitteram Diagnostic Laboratories, Inc., San Diego, CA, USA. All other reagents in experiments were commercially available analytical reagents.

### 3.2. Preparation of CTSH Sponge

Sepia ink was extracted according to literature [14]. 2% chitin and 1% sepia ink hydrogel-like was obtained by dissolving chitin and sepia ink powder in a 8% NaOH and 4% Urea solution at −40 °C for 48 h. After that, the hydrogel-like was filtered through 300 silk bolting cloth to ensure its uniformity. Then the hydrogel-like was washed with deionized water to neutral. At last, the neutral hydrogel-like was freeze-dried to get CTSH sponge. The thickness of the sponge can be controlled by the amount of chitin and sepia ink glue. 3 mm thickness of the sponge was prepared in this experiment. CTSH sponge was sealed and stored after sterilized by Cobalt-ray at 6 °C.

### 3.3. Animal Experiments

Animal experiments were conducted 5 days later when the mice adapted to the rearing environment. All of the mice have free access to food and water, and to ensure adequate sunlight and moderate temperature. All mice were equally divided into experimental group and control group. Each group consisted of 30 male mice and 30 female mice. 3 male and 3 female mice were placed into a unit from each group at testing points: 2 day, 1 week, 2 week, 4 week, 6 week, 8 week, 12 week, 16 week, 20 week and 24 week. The animal that died during the observation period or at its testing time was necropsied immediately to detect whether there were pathological changes in its viscera. Mice were anaesthetized with an intraperitoneal injection of 3% (*w/v*) pentobarbital sodium (1 mL/kg), then abdomen was shaved before fixing on their backs to the operating table. Afterward, disinfection on the abdomen with 75% alcohol should be finished before the chitin and sepia ink hybrid hemostatic sponge (CTSH sponge) (135 mg/kg) was implanted under the skin of the abdomen for experimental group while nothing for control group [33]. Benzylpenicillin (5 million units/kg) was intraperitoneal injected to disinfect after the abdominal cavity were closed by suture. We ensured that all animal experiments were carried out in accordance with Directive 2010/63/EU on the protection of animals used for scientific purposes.

### 3.4. Collection of Blood Sample

At each time point set before, 3 male and 3 female mice were selected randomly from both experimental group and control group, then anaesthetized with an intraperitoneal injection of 3% (*w/v*) pentobarbital sodium (1 mL/kg). Laparotomy began after the abdomen was shaved and disinfected. The intestine was moved laterally by using tampons, then abdominal aorta was exposed carefully. Blood was collected using 8# disposable vein infusion needles. 1 mL of blood was take into centrifuge tube on standing until the serum was fully precipitated. The serum was used for detection of FDP, PLG and PF4. 2 mL of the blood was collected into vacutainers tube containing heparin for the detection of blood viscosity index, including blood viscosity of low shear (BVL), blood viscosity of medium shear (BVM), blood viscosity of high shear (BVH) and plasma viscosity (PV). 3 mL of blood was taken into vacutainers with anticoagulant of citrate sodium to detect the indexes of prothrombin time (PT), thrombin time (TT), activated partial thromboplatin time (APTT), fibrinogen (FIB), antithrombinIII (AT-III) and D-dimer. FDP, PLG and PF4 indexes were determined with FDP, PLG and PF4 ELISA kits. The rest of the indexes were detected by the affiliated hospital of Guangdong medical college.

### 3.5. Statistical Analysis

Data were processed using Microsoft Excel 2010 software and the results were expressed as Mean ± SD. Significance between the mean values was calculated using standard software program (SPSS17.0, GmbH, Munich, Germany) and (*p* < 0.05) was considered significant.

## 4. Conclusions

With further research, chitin and its derivatives have been widely applied in the medical field because of their advantages. CTSH sponge, as a new kind of hemostatic material, was detected its biological safety in this study. Exactly 135 mg/kg CTSH sponge was implanted into the abdominal cavity of mice according to the dosage conversion among different animals. This study detected the effects of CTSH sponge on the blood parameters of mice including coagulation, anticoagulation, fibrinolytic and hemorheology at each testing time after CTSH sponge being absorbed and degraded *in vivo*. Results showed that there is no significant difference (*p* > 0.05) for all the indexes between experimental and control groups, which indicated that CTSH sponge, as a new kind of hemostatic material, has no negative effects on the mice blood systems. It provides an important reference for the practical application of CTSH sponge. Considering the wide research and potential application of CTSH sponge in medicine and tissue engineering ﬁeld [8,34,35,36], new animal models with more wound healing and haemostasis will be adopted in future, such as rat model of partial hepatectomy bleeding and miniature swine model of liver and spleen bleeding, from which the hemostatic effect of CTSH sponge can be accurately evaluated. More detailed information about CTSH sponge would be detected in the next step, including promoting healing effect, anti-infective effect, *etc*. To promote the application of CTSH sponge in clinical practice, further investigation should be carried out, such as considering the postoperative, clinical hemostatic effect, patients’ comfort and satisfaction, *etc*. These results would be reported subsequently in other experiments.
